# The Anti-fibrotic Effects and Mechanisms of MicroRNA-486-5p in Pulmonary Fibrosis

**DOI:** 10.1038/srep14131

**Published:** 2015-09-15

**Authors:** Xiaoming Ji, Baiqun Wu, Jingjing Fan, Ruhui Han, Chen Luo, Ting Wang, Jingjin Yang, Lei Han, Baoli Zhu, Dong Wei, Jingyu Chen, Chunhui Ni

**Affiliations:** 1 Department of Occupational Medicine and Environmental Health, School of Public Health, Nanjing Medical University, Nanjing, China; 2 Institute of Occupational Disease Prevention, Jiangsu Provincial Center for Disease Control and Prevention, China; 3 Nanjing Medical University, Affiliated Wuxi People’s Hospital, Lung Transplantation Center, Jiangsu Key Laboratory of Organ Transplantation, China

## Abstract

To identify microRNAs (miRNAs, miRs) with potential roles in lung fibrogenesis, we performed genome-wide profiling of miRNA expression in lung tissues from a silica-induced mouse model of pulmonary fibrosis using microarrays. Seventeen miRNAs were selected for validation via qRT-PCR based on the fold changes between the silica and the control group. The dysregulation of five miRNAs, including miR-21, miR-455, miR-151-3p, miR-486-5p and miR-3107, were confirmed by qRT-PCRs in silica-induced mouse model of pulmonary fibrosis and were also confirmed in a bleomycin (BLM)-induced mouse lung fibrosis. Notably, miR-486-5p levels were decreased in the serum samples of patients with silicosis, as well as in the lung tissues of patients with silicosis and idiopathic pulmonary fibrosis (IPF). In addition, as determined by luciferase assays and Western blotting, SMAD2, a crucial mediator of pulmonary fibrosis, was identified to be one of target genes of miR-486-5p. To test the potential therapeutic significance of this miRNA, we overexpressed miR-486-5p in animal models. At day 28, miR-486-5p expression significantly decreased both the distribution and severity of lung lesions compared with the silica group (*P* < 0.01). In addition, miR-486-5p had a similar effect in the BLM group (*P* < 0.001). These results indicate that miR-486-5p may inhibit fibrosis.

Silicosis, a fibrotic lung disease caused by inhalation of dust containing free crystalline silica, is one of the most widespread occupational diseases worldwide^1^,^2^. It is characterized by alveolar epithelial cell injury, inflammatory cell infiltration, fibroblast recruitment and extracellular matrix deposition and is related to idiopathic pulmonary fibrosis (IPF), a progressive and diffuse parenchymal disease with a high mortality rate^3^,^4^. IPF is a fatal disease with no known cause and no effective pharmacological treatment. Bleomycin (BLM) has been used to induce lung fibrosis, which mimics IPF, in both mouse and rat models^5^,^6^. Pulmonary fibrosis is a common characteristic of IPF and systemic sclerosis with interstitial lung disease (SSc-ILD)^7^; however, the most common histological pattern observed in IPF is usual interstitial pneumonia (UIP), while the pattern most commonly observed in SSc-ILD is nonspecific interstitial pneumonia (NSIP)^8^. There is some controversy regarding the pathophysiology of UIP and NSIP. Pulmonary fibrosis is an incurable and irreversible lung disease^9^, and new insights into the molecular mechanisms underlying the development of pulmonary fibrosis are needed to develop both novel therapeutic strategies and early diagnostic and prognostic biomarkers.

MicroRNAs (miRNAs, miRs) are short endogenous noncoding RNAs that play pivotal roles in a broad range of biological processes, including regulation of intracellular signaling pathways associated with various diseases^10^. Since the identification of the first miRNA^11^,^12^, approximately two thousands miRNAs have been characterized in humans (miRbase v21) ( http://www.mirbase.org/). miRNAs promote mRNA degradation and inhibit translation by binding to complementary sequences in mRNA 3′ untranslated regions (UTRs). Individual miRNAs target several mRNAs, which code for proteins with related functions^13^. Heterotypic miRNA-mRNA interactions also occur, allowing individual mRNA molecules to be targeted by several distinct miRNAs. Although the inhibitory effects of individual miRNAs on individual mRNAs may be modest, their combined effects on multiple mRNAs can evoke strong biological responses.

miRNAs play an important role in lung cells, where they have been implicated in cell proliferation, apoptosis, differentiation, etiology and the progression of fibrotic diseases^14^. Simone *et al.*^15^ reported that miRNA alterations drive both the acute and the late stages of radiation-induced fibrosis in a murine skin model. Approximately 10% of known miRNAs are reported to be altered in an IPF setting^16^. The down**-**regulation of miR-26a was observed in patients with IPF and mice with BLM-induced lung fibrosis^17^. The alteration of specific miRNA expression profiles can have a dramatic effect on fibrosis, suggesting that modulating miRNA expression may be a novel approach for developing innovative therapeutic strategies. We therefore investigated the role of miRNAs in pulmonary fibrosis to generate new perspectives regarding the disease’s underlying mechanisms, as well as possible diagnostic and therapeutic opportunities.

The aim of our study was to identify miRNAs with altered expression patterns in mice with silica-induced pulmonary fibrosis. Using miRNA microarrays, we identified a panel of miRNAs that were specifically altered in pulmonary fibrosis. Among these, miR-21, miR-455, miR-151-3p, miR-486-5p and miR-3107 were validated as being differentially expressed in fibrotic lungs compared with normal lungs. The dysregulation of these five miRNAs was also observed in a second mouse model of lung fibrosis, induced by BLM. Finally, miR-486-5p levels were decreased in serum samples from patients with silicosis, as well as in the lung tissues of patients with silicosis and IPF. In fibroblasts, SMAD2, a critical mediator of multiple fibrogenic associated-processes, including cell proliferation, migration, invasion and differentiation into myofibroblasts, was identified to be a target gene of miR-486-5p. Notably, administering miR-486-5p ameliorated experimental lung fibrosis in mice exposed to either silica or BLM.

## Results

To gain an overview of the changes in miRNA expression that occur in silica-induced pulmonary fibrosis, we performed a miRNA microarray with total RNAs isolated from mouse lung tissues harvested on days 0, 3, 7, 14, 28 and 56 after the silica challenge. Figure S1 shows photomicrographs of H&E-stained lung tissues (200×) after treatment with either silica or BLM. In total, 1,111 miRNAs were expressed at levels above background, whereas 259 miRNAs underwent at least a twofold change in expression for at least one time point after treatment. A hierarchical clustering analysis of these 259 miRNAs using GeneSpring software is shown in Figure S2A. The expression of 62 miRNAs was altered compared with day 0 for at least three of the five endpoints, including 33 up-regulated (ratio ≥2) miRNAs and 29 down-regulated (ratio ≤0.5) miRNAs (Figure S2B).

**Figure 1:**
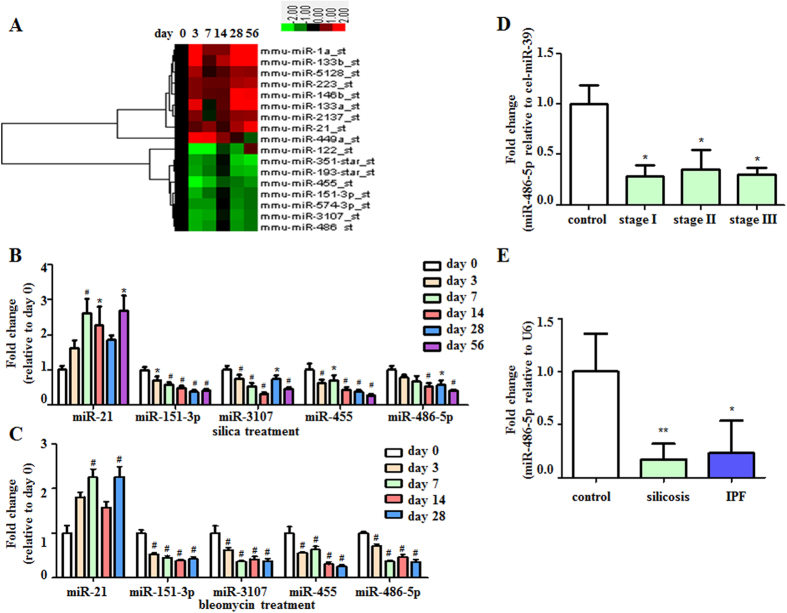
MiR-486-5p is down-regulated in the lung tissues of patients with silicosis and IPF, as well as serum from patients with silicosis. (**A**) A heat map representing the differentially expressed microRNAs in the lungs of the C57BL/6 mice in response to silica at the indicated time points. The up-regulated microRNAs are indicated in progressively brighter shades of red, and the down-regulated microRNAs are indicated in progressively brighter shades of green. (**B**) Real-time PCR validation of the microarray results from the lungs of mice with silica-induced pulmonary fibrosis. The mice were injected intratracheally with either saline or silica (50 mg/kg of silica in 0.05 ml of sterile saline) and sacrificed on days 0, 3, 7, 14, 28 and 56 after injection (n = 6 mice in each group). The data are expressed as the mean ± SD. *P < 0.05, ^#^*P* < 0.01. (**C**) The mice were injected intratracheally with either saline or bleomycin (1.5 U/kg of bleomycin in 0.05 ml of sterile saline) and sacrificed on days 0, 3, 7, 14 and 28 after injection. The five validated miRNAs were consistent with the microarray results from the BLM-induced pulmonary fibrosis model as determined via a real-time PCR analysis. U6 was used as an internal control (n = 6 mice in each group). The data are expressed as the mean ± SD. *P < 0.05, ^#^*P* < 0.01. (**D**) The serum concentrations of the five validated miRNAs were measured in 60 patients with silicosis (n = 20 per stage I, II, III) and 20 normal control subjects by qRT-PCR. The data are expressed as the mean ± SD. *P < 0.05. (**E**) miR-486-5p expression in the lungs of patients with either silicosis (n = 5) or IPF (n = 5) was measured by qRT-PCR. The data are expressed as the mean ± SD. *P < 0.05 compared with the normal control lungs (n = 2).

To validate the results of our miRNA profiling experiment, we confirmed the differential expression of these miRNAs via real-time quantitative reverse transcription polymerase chain reactions (qRT-PCRs). Seventeen miRNAs with raw expression values greater than 100 were selected from among the 62 miRNA molecules with altered expression levels (Fig. 1A). Five miRNAs, miR-21, miR-455, miR-151-3p, miR-486-5p and miR-3107, were validated to be dysregulated in the lung tissues from mice with silica-induced fibrosis (n = 6 per group) (Fig. 1B). Because fibrotic diseases may share common pathogenic mechanisms^18^,^19^,^20^, we sought to validate our findings in another experimental model of pulmonary fibrosis (induced by BLM), as well as in human fibrotic diseases. We found that miR-21, miR-455, miR-151-3p, miR-486-5p and miR-3107 were differentially expressed in a BLM-induced mouse model of pulmonary fibrosis (Fig. 1C). In serum samples obtained from patients with silicosis, miR-486-5p expression was significantly decreased in all cohorts (stages I, II and III, *P* < 0.05) compared with the control subjects (Fig. 1D). The lung tissues obtained from the patients with either silicosis or IPF also exhibited decreased miR-486-5p expression (Fig. 1E).

**Figure 2:**
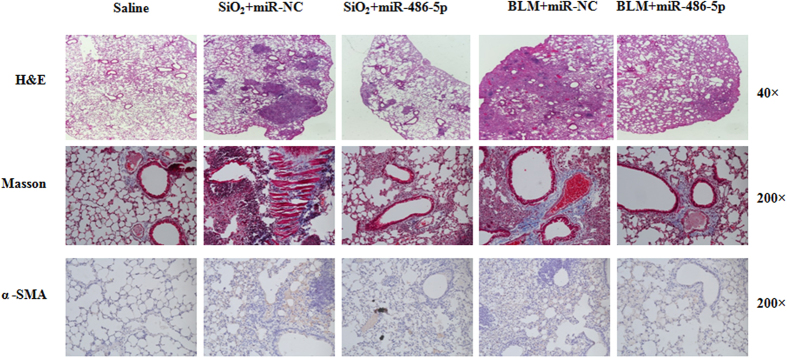
Increased miR-486-5p expression attenuates experimental pulmonary fibrosis. (**A**) A schematic of the silica- and bleomycin-induced models of lung fibrosis, indicating time points for the delivery of miR-486-5p mimics. For miR-486-5p treatment, control mimics plus silica/bleomycin (10 mg/kg of agomiR-NC plus either 50 mg/kg of silica or 1.5 U/kg of bleomycin in 50 μl of saline) and the miR-486-5p mimics plus silica/bleomycin (10 mg/kg of agomiR-486-5p plus either 50 mg/kg of silica or 1.5 U/kg of bleomycin in 50 μl of saline) were administered intratracheally. Either 4 mg/kg of agomiR-486-5p or agomiR-NC was subsequently injected via the tail vein on a weekly basis (n = 6 for each group). The mice were sacrificed 28 days after either silica or bleomycin administration. (**B**) The lung sections were stained using H&E (40 × magnification) and Masson’s trichrome (200 × magnification), as described in the Materials and Methods section. Immunohistochemistry assays were performed to measure α-SMA expression (200 × magnification). The results shown are representative of three independently performed experiments.

Having demonstrated that miR-486-5p was decreased in two mouse models of lung fibrosis as well as human silicosis and IPF, we next explored whether restoring miR-486-5p expression may have therapeutic potential. To address this question, we administered either silica or BLM together with either control mimics or miR-486-5p mimics to mice intratracheally. At 28 days after treatment, the mice that were treated with miR-486-5p exhibited decreased lung fibrosis as determined by histological analyses of the lungs and Masson’s trichrome assays for collagen deposition (Fig. 2). miR-486-5p overexpression also reduced α-SMA expression in the fibrotic lungs, which suggests that miR-486-5p diminishes myofibroblast differentiation (Fig. 2). Additionally, as shown in Table 1, both the severity and the distribution of the lung lesions decreased following miR-486-5p administration compared with the silica group (*P* < 0.01). miR-486-5p overexpression had a similar effect in the BLM group (*P* < 0.001).

**Figure 3:**
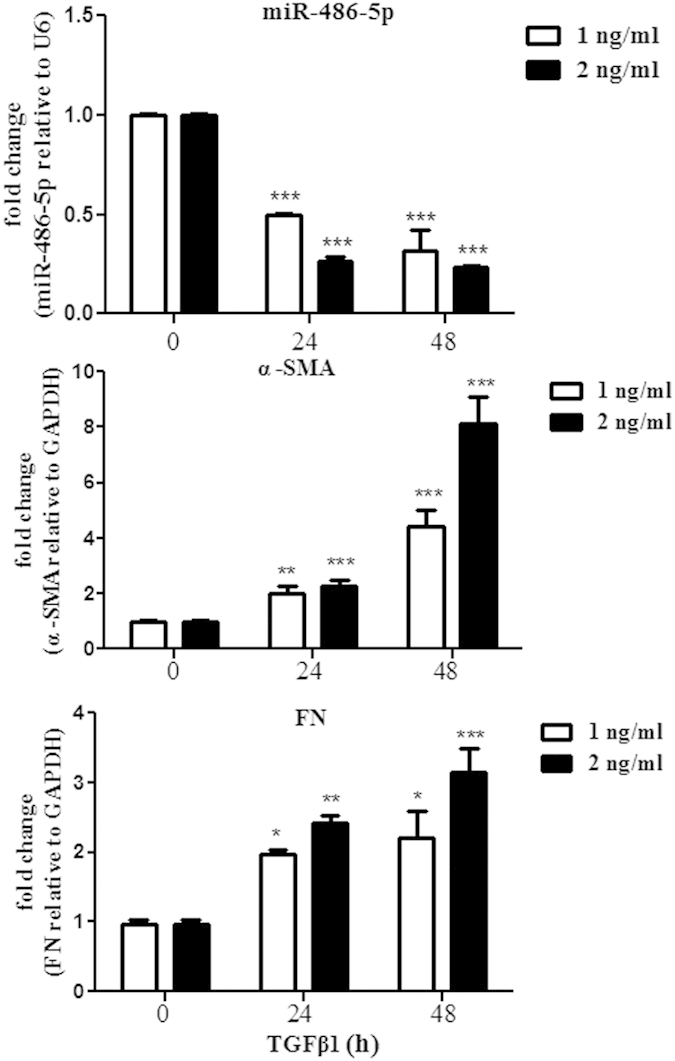
TGF-β1 down-regulates miR-486-5p expression in lung fibroblasts. Mouse fibroblasts (NIH/3T3 cells) were treated with TGF-β1 (either 1 ng/ml or 2 ng/ml) for 0, 24, or 48 h. The RNA was isolated, and the levels of miR-486-5p, fibronectin (Fn) and α-SMA were determined by real-time PCR. The values are presented as the mean ± SD. The experiments were performed 3 times, with similar results. *P < 0.05, **P < 0.01, ***P < 0.001 vs. time 0.

Uniquely, miR-486-5p was decreased not only in fibrotic lung tissues but also in serum from patients with silicosis. Moreover, treating mouse fibroblasts (NIH/3T3 cells) with TGF-β1 resulted in increased mRNA expression of the myofibroblast differentiation markers Fn and α-SMA and decreased levels of miR-486-5p (Fig. 3). All these data indicate that down-regulation of miR-486-5p contributes to pulmonary fibrosis.

**Figure 4:**
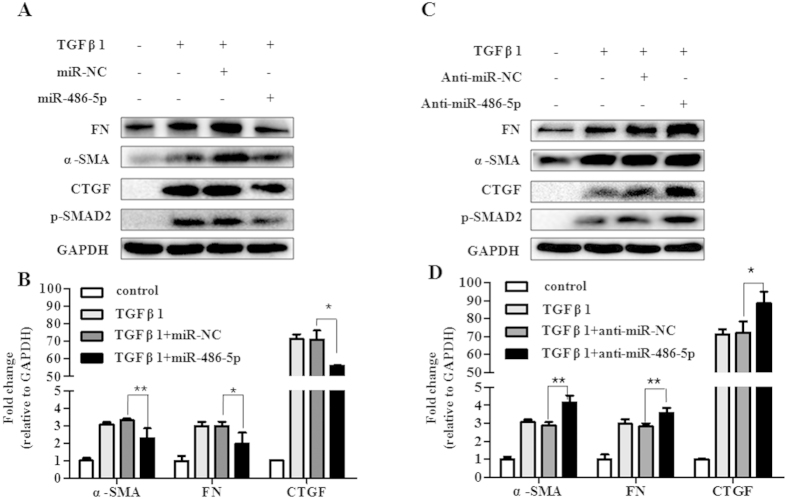
miR-486-5p regulates fibrogenesis in fibroblasts. (**A**) Mouse fibroblasts (NIH/3T3 cells) were transfected with either 50 nM of control mimics (miR-NC) or 50 nM of the miR-486-5p mimics. At 2 d after transfection, the cells were serum-starved by incubating in medium containing 0.1% FCS for 1 d, followed by treatment either with or without 2 ng/ml TGF-β1 for 1 d. The levels of α-SMA, Fn, p-SMAD2, CTGF and GAPDH were determined via Western blotting. Three independent experiments were performed. (**B**) The experiments were performed as in (**A**) The levels of α-SMA, Fn, CTGF and GAPDH were determined via real-time PCR. *n* = 3, and the values are expressed as the means ± SD. *P < 0.05, **P < 0.01 and ***P < 0.001. (**C**) Normal mouse fibroblasts (NIH/3T3 cells) were transfected with either 100 nM of the control inhibitors (anti-miR-NC) or 100 nM of the miR-486-5p inhibitors (anti-miR-486-5p). At 2 d after transfection, the cells were starved by incubating in medium containing 0.1% FCS for 1 d, followed by treatment either with or without 2 ng/ml TGF-β1 for 1 d. The levels of α-SMA, Fn, p-SMAD2, CTGF and GAPDH were determined via Western blotting. Three independent experiments were performed. (**D**) The experiments were performed as in **C**. The levels of α-SMA, Fn, CTGF and GAPDH were determined via real-time PCR. n = 3, and the values are expressed as the mean ± SD. *P < 0.05, **P < 0.01 and ***P < 0.001.

To further explore if miR-486-5p down-regulation is required for fibrogenesis in mouse fibroblasts, NIH/3T3 cells were transfected with either miR-486-5p mimics or control mimics (miR-NC) for 48 hours and then treated with TGF-β1 for another 24 hours. As shown in Fig. 4A,B, over-expression of miR-486-5p in the fibroblasts decreased TGF-β1-induced α-SMA, Fn and CTGF expression at both the protein and the mRNA levels compared with the control mimics (Fig. 4A,B). These data suggest that decreased miR-486-5p is required for fibrogenesis. To further determine if miR-486-5p down-regulation is sufficient to promote fibrogenesis in mouse fibroblasts , we knocked down miR-486-5p expression in mouse fibroblasts (NIH/3T3 cells). We observed that treatment with TGF-β1 up-regulated the expression of α-SMA, Fn and CTGF and that this expression was enhanced in the anti-miR-486-5p-transfected cells (Fig. 4C,D). These data suggested that decreased miR-486-5p is sufficient to promote fibrogenesis in mouse fibroblasts.

**Figure 5:**
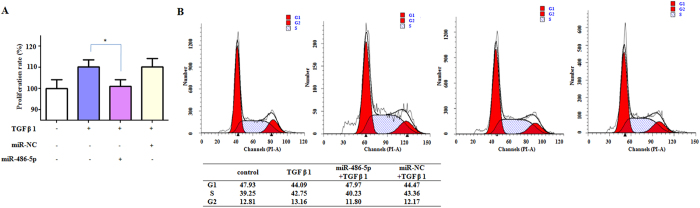
miR-486-5p represses the TGF-β1-induced proliferation of fibroblasts. NIH/3T3 cells were transfected with either the miR-486-5p mimics or miR-NC. At 48 hours post-transfection, the cells were treated with TGF-β1 for 24 hours. (**A**) The CCK-8 assay demonstrates significantly increased cell numbers in the TGF-β1 treatment group compared with the control group, and decreased cell numbers when miR-486-5p was administered compared with the TGF-β1 treatment group. *P < 0.05. (**B**) Flow cytometric analysis shows that treatment with TGF-β1 increases the numbers of cells in S phase and decreases the numbers of cells in G1 phase (n = 3). *P < 0.05 compared with the control group. The miR-486-5p mimics restored the effects on the cell cycle (n = 3).

We also sought to illustrate whether miR-486-5p interferes with TGF-β1-induced proliferation in NIH/3T3 cells. At 48 hours post-transfection with miR-486-5p, the cells were treated with TGF-β1 for 24 hours. A CCK-8 assay demonstrated significant increases in cell numbers in the TGF-β1 treatment group compared with the control group. However, decreased cell numbers were observed in the cells transfected with the miR-486-5p mimics but not in the cells transfected with miR-NC, indicating that miR-486-5p successfully suppressed the TGF-β1-induced proliferation of fibroblasts (Fig. 5A). Next, we examined the effects of miR-486-5p on cell-cycle progression in NIH/3T3 cells and observed a significant increase in S phase and a decrease in G1 phase after TGF-β1 treatment. These effects were restored by miR-486-5p, which suggests a role for miR-486-5p in repressing TGF-β1-induced proliferation by increasing the number of cells in S phase and decreasing the cell-cycle arrest (Fig. 5B).

**Figure 6:**
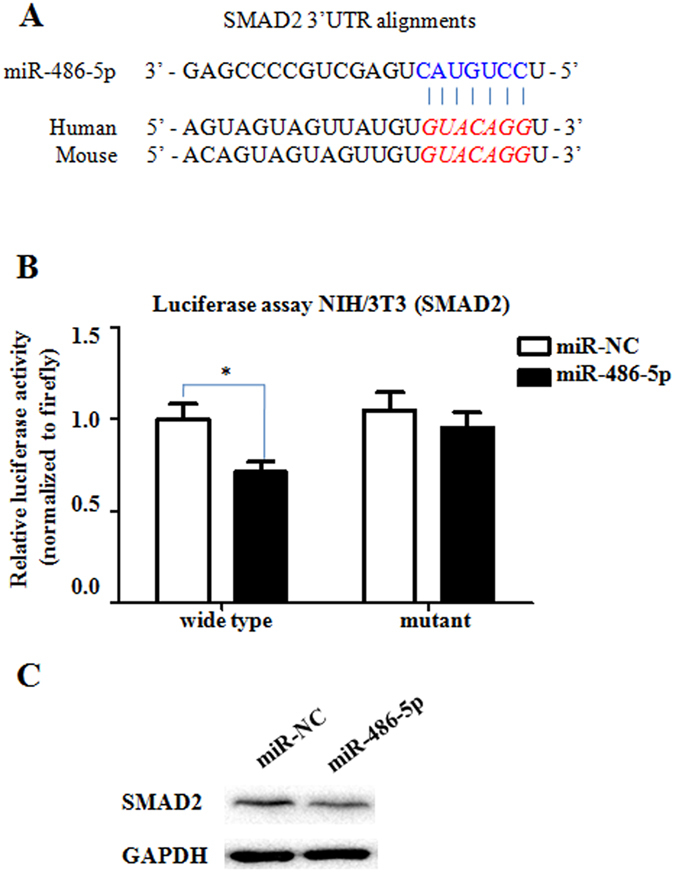
SMAD2 is a direct target of miR-486-5p. (**A**) The position of the miR-486-5p target site in the SMAD2 3′ UTR and the sequence alignment of miR-486-5p and the SMAD2 3′ UTR from various species are shown. The bases that were mutated in the psiCHECK-2 construct are italicized. (**B**) Either miR-486-5p or miR-NC was co-transfected with the human SMAD2 3′UTR-derived psiCHECK-2 construct (either wild type or mutated in the putative miR-486-5p seed region) into NIH/3T3 cells. The cells were harvested two days after transfection, and the luciferase activity was analyzed. The Renilla luciferase activity was normalized to the firefly luciferase activity. The values are presented as the mean ± SD. The experiments were performed three times with similar results. *P < 0.05. (**C**) The Western blotting analysis demonstrates the down-regulation of SMAD2 protein expression after transfection of the mouse fibroblasts (NIH/3T3 cells) with miR-486-5p. One representative experiment out of three is depicted.

In addition, the seed sequence of miR-486-5p within the 3′UTR sequence of mouse SMAD2 was predicted to be a potential conserved binding site (Fig. 6A). To determine whether miR-486-5p could directly regulate SMAD2, we cloned part of the 3′UTR of SMAD2 into a luciferase reporter construct (the psiCHECK-2 reporter). A 3′UTR mutant with mutations in the predicted miR-486-5p site was also cloned into a psiCHECK-2 reporter. The constructs were subsequently transfected into NIH/3T3 cells with either miR-486-5p mimics or control mimics (miR-NC)(Fig. 6B). Co-transfection with miR-486-5p significantly reduced the normalized luciferase activity for the reporter containing the wild type, but not the mutant, suggesting that SMAD2 is directly regulated by miR-486-5p. Furthermore, transfecting NIH/3T3 cells with miR-486-5p decreased SMAD2 protein expression (Fig. 6C), demonstrating that SMAD2 is targeted by miR-486-5p directly.

## Discussion

Crucial new insights into the pathogenesis, diagnosis, treatment and prognosis of numerous human diseases, including tissue fibrosis, have been provided by genome-wide approaches to miRNA expression profiling^21^,^22^,^23^,^24^. The application of such approaches in IPF has demonstrated that let-7d, miR-21 and miR-199a-5p make critical contributions to pulmonary fibrosis^25^,^26^,^27^. In the present study, we identified a specific miRNA profile associated with silica-induced pulmonary fibrosis and confirmed the role of dysregulated miRNAs in the pathogenesis of both silica- and BLM-induced lung fibrosis. miR-21, miR-455, miR-151-3p, miR-486-5p and miR-3107 were differentially expressed in mouse fibrotic lung tissues. Additionally, miR-486-5p expression was decreased in serum samples from patients with silicosis, as well as the lung tissues of patients with either silicosis or IPF, compared with healthy donors. Therefore, miR-486-5p may represent a primary pathogenic mechanism underlying the development of lung fibrosis.

miR-486-5p is generated by processing intronic RNA from the *Ank1* gene^28^. The *Ank1* gene encodes an ankyrin repeat domain protein that links the cytoskeleton to the plasma membrane and is transcribed as either a short (heart muscle- and skeletal muscle-enriched)^29^ or a long (erythroid-enriched)^30^ isoform, depending on the cell and tissue type. miR-486-5p is highly conserved among mammals, and no miR-486 sequence has been identified within the genomes of non-mammalian species such as fishes or birds, even though these species contain the *Ank1* gene sequence^31^. miR-486 is located at Chr:8p11, a region of frequent genomic loss in multiple cancers^32^. The human miR-486-5p precursor generates two mature miRNAs: miR-486-5p and miR-486-3p. Decreased expression of miR-486-5p was observed in tumor tissues from patients with lung, colon, melanoma and gastric cancer^33^,^34^,^35^,^36^, and miR-486-3p dysregulation has been detected in both pancreatic and esophageal cancer^37^,^38^. In the present study, decreased miR-486-5p expression was linked to the progression of pulmonary fibrosis in both humans and mice. Decreased expression of miR-486-5p was observed in both silica- and BLM-induced mouse models of lung fibrosis, and miR-486-5p expression was decreased in tissue samples from patients with silicosis and IPF . Moreover, our data indicate that over-expression of miR-486-5p attenuated pulmonary fibrosis in mice and repressed TGF-β1-induced fibrogenesis in NIH/3T3 cells. These findings demonstrate that miR-486-5p has a strong anti-fibrotic activity in lung tissues and may be a novel target in the treatment of pulmonary fibrosis.

Considerable evidence suggests that miRNAs play critical roles in the progress of lung fibrosis and may represent promising therapeutic targets for fibrotic lung diseases. For example, Liu *et al.* found that miR-21 is over-expressed in the lungs of mice with BLM-induced fibrosis and patients with IPF^25^. Administration of miR-21 antisense diminished the severity of the experimental lung fibrosis observed in the mice^25^. Pandit *et al.* demonstrated that let-7d expression was significantly reduced in the lungs of patients with IPF and that the number of epithelial cells expressing let-7d correlated with pulmonary function^26^. Furthermore, miR-31, miR-200 and miR-29 each play an anti-fibrotic role in the lungs^39^,^40^,^41^. However, until now there has been no evidence that miR-486-5p decreases lung fibrosis. The present study is the first to show that administration of miR-486-5p mimics may help to attenuate the fibrotic processes associated with lung disease.

Pulmonary fibrosis is characterized by aberrant fibroblast proliferation and increased deposition of extracellular matrix (ECM)^42^. When injured, lung fibroblasts undergo phenotypic modulation to become α-SMA positive myofibroblasts, which is a crucial step in collagen secretion and the repair process that occurs following lung injury. The myofibroblast proliferation and collagen deposition at the site of injury ensure adequate scar formation, which helps to maintain the structural integrity and function of the alveoli^43^. Activation of the TGF-β1 pathway is a significant event in the fibrogenic response, as it contributes to the differentiation of pulmonary fibroblasts into myofibroblasts and triggers the synthesis of ECM proteins^44^. In the process of fibrosis, TGF-β1 interacts with a complex of transmembrane serine/threonine kinase receptors (TGF-βR II/TGF-βR I), resulting in phosphorylation of the transcription factors Smad2/Smad3, which form a complex with Smad4. This heteromeric complex translocates to the nucleus, where it interacts with DNA, transcription factors, coactivators, and corepressors to modulate profibrotic processes. By contrast, SMAD7 negatively regulates TGF-β1 signaling by competing with Smad2 and Smad3 for TGF-βRI binding^45^,^46^. SMAD2 is a key signal transducer and transcription factor in the TGF-β1 signaling pathway. Our data indicate that miR-486-5p has an impact on the development of lung fibrosis partly by targeting SMAD2, which is one of downstream molecules of the TGF-β1 signaling pathway. Moveover, the TargetScan database predicts that Col6α6 is a potential target of miR-486-5p. Accordingly, we observed that miR-486-5p post-transcriptionally regulates Col6α6 in lung fibroblasts (Figure S3). These results support the hypothesis that miR-486-5p exerts its anti-fibrotic effects by suppressing collagen synthesis and targeting SMAD2 directly.

There are several possible reasons for the down-regulation of miR-486-5p that we observed in fibrotic lung tissues. One potential explanation is that miR-486-5p is located on chromosome 8p11.21, a genomic region containing several potential tumor-suppressor genes that is frequently deleted in a variety of cancers^47^. The loss of heterozygosity in this region may result in the down-regulation of miR-486-5p. Additionally, miR-486-5p is located within a CpG island on chromosome 2q35, so epigenetic silencing via either DNA methylation or histone deacetylation may also be responsible for miR-486-5p down-regulation^33^. Nevertheless, additional studies are required to evaluate the causes of miR-486-5p dysregulation in lung fibrogenesis.

miRNAs normally have multiple targets^48^. Therefore, inhibition of SMAD2 may not be the sole mechanism by which miR-486-5p exerts its anti-fibrogenic effects. For example, *PTEN*, a major negative regulator of the PI3-kinase pathway, which regulates growth, survival and proliferation, is targeted by miR-486-5p in muscle cells^28^. Furthermore, *OLFM4* was recently proposed to be a biologically relevant miR-486-5p target in gastric cancer^49^. Additionally, the down-regulation of miR-486-5p was reportedly responsible for both tumor progression and metastasis by relieving the inhibition of protumorigenic ARHGAP5 in lung cancer^33^. Insulin-like growth factor 1 (IGF-1), a positive regulator of the Akt-signaling pathway, is down-regulated by miR-486-5p in muscle cells directly^28^. IGF-1, which has been shown to stimulate differentiation of fibroblasts into a myofibroblast phenotype in a soft matrix environment, exerts a mild effect on α-SMA stress fiber organization in mouse lung fibroblasts^50^. Increased IGF-1expression was observed in the lung tissues of BLM-treated C57BL/6 mice^5^. The studies mentioned above suggest that IGF-1may also be associated with the anti-fibrotic effects of miR-486-5p. Consequently, given its broad involvement in fibrotic signaling events, targeting miR-486-5p in patients with silicosis and IPF may represent a superior therapeutic strategy compared with approaches aimed at a single pathway. Our data highlight the ability of miRNAs to fine**-**tune various cellular and developmental events, as opposed to abolishing the expression of a single protein^51^.

Our study indicates that miR-486-5p expression is frequently reduced in lung fibrotic diseases and may act as an anti-fibrotic effector in the development of pulmonary fibrosis. The results point to a novel approach for treating fibrotic diseases such as silicosis and IPF, for which cures have long proven elusive, by using therapeutics that specifically target miR-486-5p.

## Materials and Methods

### Cell culture, reagents and antibodies

Mouse fibroblast cells (NIH/3T3) were obtained from the American Type Culture Collection (ATCC, Manassas, VA, USA). The cells were frozen at an early passage and cultured for a maximum of eight passages. The cells were maintained at 37 °C with 5% v/v CO_2_ in Dulbecco’s modified Eagle’s medium (DMEM, Life Technologies/Gibco, Grand Island, NY) supplemented with 10% fetal calf serum (FCS, Life Technologies/Gibco, Grand Island, NY), 100 U/ml penicillin G and 100 μg/ml streptomycin (Life Technologies/Gibco, Gaithersburg, MD). Recombinant TGF-β1 was obtained from Sigma-Aldrich. The rabbit anti-SMAD2 monoclonal antibody (ab33875), rabbit anti-α-SMA monoclonal antibody (ab32575), rabbit anti-fibronectin monoclonal antibody (ab32419) and rabbit anti-p-SMAD2 monoclonal antibody (ab188334) were obtained from Abcam. The rabbit anti-CTGF monoclonal antibody (E-5) was obtained from Santa Cruz. The anti-GAPDH (13E5) monoclonal antibody was obtained from Cell Signaling Technology. The GAPDH levels served as internal controls.

### Animals, Induction of Pulmonary Fibrosis and Treatment

All animal procedures were conducted in accordance with humane animal care standards approved by the Nanjing Medical University Ethics Committee (Nanjing, China). Male C57BL/6 mice (6-8 weeks of age; 18-20g) were obtained from SLAC China (Shanghai, China) and maintained under specific pathogen-free conditions. The animals were acclimated to the environment for 1 week prior to treatment.

#### Silica-induced Mouse Model of Lung Fibrosis

Silica-induced pulmonary fibrosis was induced by intratracheal administration of crystalline silica particles (Sigma Aldrich, USA). The animals were anesthetized with sodium pentobarbital (Dainippon Sumitomo Pharma, Osaka, Japan), the tracheas were exposed, and 50 mg/kg of silica or 0.05 ml of sterile saline were administered intratracheally. The mice were sacrificed for lung collection on days 0, 3, 7, 14, 28 and 56 after silica administration (n = 6 for each time point). The miRNA microarray expression analyses were performed on one mouse per group, and the other validation experiments were performed using 6 mice per group.

#### Bleomycin-induced Mouse Model of Lung Fibrosis

The mice were administered with BLM (Nippon Kayaku, Tokyo, Japan) intratracheally at a dose of 1.5 U/kg dissolved in a total of 0.05 ml of sterile saline. The control groups were treated with 0.05 ml of sterile saline using the same method. The mice were killed on days 0, 3, 7, 14 and 28 (n = 6 for each time point). The expression analyses were performed using 6 mice per group.

#### Treatment

The cholesterol-conjugated miR-486-5p mimics and negative controls (agomiR-486-5p and agomiR-NC, respectively) were purchased from RiboBio Co., Ltd. (Guangzhou, China). The control mimics plus silica/BLM (10 mg/kg agomiR-NC plus either 50 mg/kg silica or 1.5 U/kg BLM in 50 μl saline) and the miR-486-5p mimics plus silica/BLM (10mg/kg agomiR-486-5p plus either 50 mg/kg silica or 1.5 U/kg BLM in 50 μl saline) were administered intratracheally. Either 4 mg/kg agomiR-486-5p or agomiR-NC was subsequently injected via the tail vein on a weekly basis (n = 6 for each group). The mice were sacrificed 28 days following either silica or BLM administration.The experimental design is shown in Figure S4.

### Human lung tissue and serum

Five silicosis and 5 IPF lung tissue samples were obtained from either biopsy specimens or lung tissue samples from patients with either silicosis or IPF who underwent a pulmonary transplant. Two control samples resected from the lungs of healthy donors were obtained from the Wuxi People’s Hospital. Serum samples from 60 patients at different stages of silicosis (stages I-III) and 20 control subjects were also collected. Written informed consent was obtained from all subjects. The research protocol was approved by the Institutional Review Board of Nanjing Medical University, and all experiments were completed in accordance with previously approved guidelines.

### Histopathology

The mouse lungs were inflated with a neutral buffered formalin solution overnight and embedded in paraffin before sectioning into 5 μm-thick slices. The sections were stained with hematoxylin and Masson’s trichrome staining to assess the degree of fibrosis. An experienced pathologist reviewed the histological sections.

The degree of alveolar wall thickening, cellular proliferation, inflammatory lesions and collagen deposition, as well as the extent of fibrotic damage, served as the basis for the assessment of the structural changes that occurred in the tissues. Such alterations were divided into different grades of severity and distribution. The same grading system was utilized for each group of animals^52^.

Lesion severity was graded as follows: 0 = nothing/zero, 1 = marginal, 2 = slight, 3 = moderate, 4 = severe and 5 = very severe. Lesion distribution was graded as follows: 0 = absent, 1 = rare/occasional (10% ofthe lung area), 2 = sparse/limited (10%–25% of the lung area), 3 = moderate (25%–50% of the lung area), 4 = extensive/widespread(50%–75% of the lung area) and 5 = veryextensive/predominant (over 75% of the lung area).

### miRNA microarray

Total RNA was isolated from the mouse lungs harvested on days 0, 3, 7, 14, 28 and 56 after silica administration (one mouse per group) using TRIzol (Invitrogen, Carlsbad, CA, USA). The pathology of the lung tissues was carefully evaluated (Figure S1). miRNA profiling was performed using an Affymetrix miRNA microarray service (miRNA 3.0) from CapitalBio (Beijing, China). The experimental data and microarray design have been deposited in the National Center for Biotechnology Information-Gene Expression Omnibus (GSE54463). The differentially expressed miRNAs from the mice at different time points after silica treatment were defined as the base of the fold change (log_2_) compared to the control samples. The miRNAs exhibiting an expression fold change (log_2_) greater than 1 or less than -1 were deemed to be differentially expressed. We validated a series of abundantly and differentially expressed miRNAs via quantitative reverse transcription-PCR.

### RNA extraction and quantitative reverse transcription-PCR.

#### Mature miRNA expression

The expression of mature miRNAs was assayed using TaqMan MicroRNA Assays (Applied Biosystems, Foster City, CA) specific for *hsa-miR-486* (ID 001278), *hsa/mmu-miR-21a* (ID 000397), *hsa-miR-455* (ID 001280), *hsa-miR-151-3p* (ID 002254), *mmu-miR-1a* (ID 002222), *mmu-miR-133b* (ID 002247), *mmu-miR-5128* (ID 462199_mat), *mmu-miR-223* (ID 002295), *mmu-miR-146b* (ID 001097), *mmu-miR-133a* (ID 001637), *mmu-miR-449a* (ID 001030), *mmu-miR-122* (ID 002245), *mmu-miR-351-3p* (ID 464446_mat), *mmu-miR-193a-5p* (ID 002577), *mmu-miR-151-3p* (ID 001190), *mmu-miR-574-3p* (ID 002349), *mmu-miR-3107/486* (ID 001278). qRT-PCR was performed using an ABI 7900 cycle detection system. Each sample was analyzed in triplicate. The data were analyzed via the 2^−△△CT^ method, and the expression of the target miRNAs was normalized to *cel-miR-39* (ID 000200) in the serum samples and U6 in the lung tissue samples (ID 001973).

#### Gene expression

The expression levels of mouse fibronectin (Fn), α-SMA, CTGF and GAPDH were analyzed using a TaqMan MicroRNA Assay (Applied Biosystems) according to the manufacturer’s instructions. The primer sequences (forward and reverse) were as follows: Fn, TCTGGGAAATGGAAAAGGGGAATGG and CACTGAAGCAGGTTTCCTCGGTTGT; α-SMA, GACGCTGAAGTATCCGATAGAACACG and CACCATCTCCAGAGTCCAGCACAAT; CTGF, GCGAAGCTGACCTGGAGGA and CGCACGAGTGGTGGTTCTGTGCG; and GAPDH, CGACTTCAACAGCAACTCCCACTCTTCC and TGGGTGGTCCAGGGTTTCTTACTCCTT. Real-time PCR was performed usingTaqMan Gene Expression Master Mix (Applied Biosystems) and an ABI7900HT real-time PCR machine. The expression levels were evaluated via the 2^−△△CT^ method, and the expression levels of the target mRNAs were normalized to GAPDH.

### Transfection and luciferase assays

The mature miR-486-5p mimics (miR-486-5p), control mimics (miR-NC), miR-486-5p inhibitors (anti-miR-486-5p) and control inhibitors (anti-miR-NC) were designed and synthesized by RiboBio Co., Ltd. (Guangzhou, China). The cells were seeded in medium without antibiotics approximately 24 h before transfection. Oligonucleotide transfection at a final concentration of 50 nM was performed using Lipofectamine 2000 (Invitrogen, Carlsbad, CA) according to the manufacturer’s protocol. After 48 h, the cells were collected for the assays.

The molecular constructs were generated in psiCHECK-2 (Promega) by cloning into the at the XhoI and NotI restriction sites upstream of the coding sequence for Renilla luciferase using annealed oligonucleotides derived from the Smad2 3′UTR, as described below. The mutated nucleotides located within the miR-486-5p-binding site are underlined. The NIH/3T3 cells were plated in 24-well plates and co-transfected using Lipofectamine 2000 (Invitrogen) with 200 ng of psiCHECK-2 and either miR-486-5p or miR-NC at different concentrations. Forty-eight hours after transfection, the firefly and Renilla luciferase activity was measured using the Dual-Glo Luciferase assay (Promega).

### Immunoblotting assay

Nuclear extracts were obtained using a lysis buffer (M-PER protein extraction reagent for the cells, T-PER protein extraction reagent for the tissues) supplemented with a 1:100 protease inhibitor cocktail (Pierce). The protein concentrations were measured using a Bradford Protein Assay kit (Bio-Rad) according to the manufacturer’s instructions. The proteins (10 mg per sample) were resolved on an SDS-PAGE gel and transferred to nitrocellulose membranes (GE Healthcare). The membranes were blotted with specific antibodies (SMAD2, α-SMA, FN, p-SMAD2 or GAPDH) overnight at 4 °C. The membranes were subsequently washed with TBST for 30 minutes at room temperature, incubated with horseradish peroxidase-conjugated secondary antibodies for 60 minutes, and washed a second time with TBST for 30 minutes. Protein expression was detected using Amersham ECL Western Blotting Substrate (GE Healthcare).

### Immunohistochemistry

Five-μm-thick paraffin-embedded sections were deparaffinized with xylene (twice for 5 minutes) before being rehydrated in water using an ethanol gradient. After washing with water, antigen retrieval was performed in a steamer using citrate buffer (pH 6.0; DAKO) for 20 minutes, and the samples were then cooled to room temperature. The sections were then washed with TBST buffer and incubated with 3% H_2_O_2_ for 10 minutes and blocked with avidin/biotin blocker and serum-free blocking reagent. The sections were subsequently incubated with rabbit anti-α-SMA antibody (Abcam) overnight at 4 °C. The DAB substrate system (DAKO) was used to detect the immunohistochemical staining. 3DHISTECH pannoramic viewer was used for viewing digital slides.

### Cell proliferation assay

The NIH/3T3 cells (2.5 × 10^3^/well) were seeded in 96-well plates in triplicate. The cells were serum-starved the following day and transfected with 50 nM of either the miR-486-5p mimics or the negative controls. Forty-eight hours after transfection, the cells were treated with 1 ng/ml TGF-β1 for 24 hours. Cell proliferation was measured using a Cell Counting Kit (CCK-8, Dojindo, Japan). The cell cycle was assessed using propidium iodide (PI) (Sigma, USA) staining, followed by a flow cytometric analysis, according to the manufacturer’s protocol. Briefly, at 48 h post-transfection with either the miR-486-5p mimics or the control mimics (50 nM), the NIH/3T3 cells were collected via trypsinization and washed with phosphate-buffered saline (PBS). For the cell-cycle analysis, the cells were fixed with 75% ethanol and stored at 4 °C overnight. The following day, the cells were washed with PBS, treated with RNase A (50 μg/ml), and stained with propidium iodide (PI) (50 μg/ml) for 30 min in the dark. The cells were subsequently analyzed via flow cytometry (FACSCalibur, Becton-Dickinson). The cells in the G0/G1, S, and G2/M phases were quantified using FlowJo 7.6.2 (Treestar).

### Statistical Analysis

The data are presented as mean values, and the error bars indicate standard deviation (SD). The quantitative variables between two groups were compared using the independent-samples *t* test, and among more groups via one-way analysis of variance (ANOVA) using Dunnett’s test (day 0 as the control group). *P* < 0.05 was considered statistically significant.

## Additional Information

**How to cite this article**: Ji, X. *et al.* The Anti-fibrotic Effects and Mechanisms of MicroRNA-486-5p in Pulmonary Fibrosis. *Sci. Rep.* **5**, 14131; doi: 10.1038/srep14131 (2015).

## Supplementary Material

Supplementary Information

## Figures and Tables

**Table 1:** Alterations in lesion severity and distribution in lungs in response to treatment with miR-486-5p.

Group	Lesion severity grade						Average severity grade	Lesion distribution grade						Average distribution grade
	0	1	2	3	4	5		0	1	2	3	4	5	
saline	6						—	6						—
silica				3	3		3.5 ± 0.5			2	3		1	3.0 ± 1.0
silica + mi		1	3	2			2.2 ± 0.8^a^		5	1				1.2 ± 0.4^a^
BLM					2	4	4.5 ± 0.8			1	2	2	1	3.5 ± 1.0
BLM + mi			4	2			2.3 ± 0.5^b^		5	1				1.2 ± 0.4^b^

^a^*P* < 0.01 significantly altered compared with the silica group (independent-samples t test).

^b^*P* < 0.001 significantly altered compared with the BLM group (independent-samples t test).
