# Supplementary material for: The Anti-fibrotic Effects and Mechanisms of MicroRNA-486-5p in Pulmonary Fibrosis
Source: Sci Rep. 2015 Sep 15;5:14131. doi: 10.1038/srep14131 (PMC4569899; doi:10.1038/srep14131)
Supplement: Supplementary Information [file srep14131-s1.pdf]

The Anti-fibrotic Effects and Mechanisms of MicroRNA-486-5p in Pulmonary Fibrosis

Xiaoming Ji[1†], Baiqun Wu[1†], Jingjing Fan[1†], Ruhui Han [1†], Chen Luo[1], Ting Wang[1], Jingjin Yang[1], Lei Han[1,2] , Baoli Zhu[2], Dong Wei[3], Jingyu Chen[3], Chunhui Ni[1,*]

1) Department of Occupational Medicine and Environmental Health, School of Public Health, Nanjing Medical University, Nanjing, China

2) Institute of Occupational Disease Prevention, Jiangsu Provincial Center for Disease Prevention and Control, China

3) Nanjing Medical University, Affiliated Wuxi Peoples Hospital, Lung Transplantation Center, Jiangsu Key Laboratory of Organ Transplantation, China

Running Title: miR-486-5p in Pulmonary Fibrosis

[†]These authors contributed equally to this work.

*Correspondence to:Department of Occupational Medicine and Environmental Health, School of Public Health, Nanjing Medical University, Nanjing 211166, China. Tel: +86 25 86868419; Fax: +86 25 86868499; Email: chninjmu@126.com

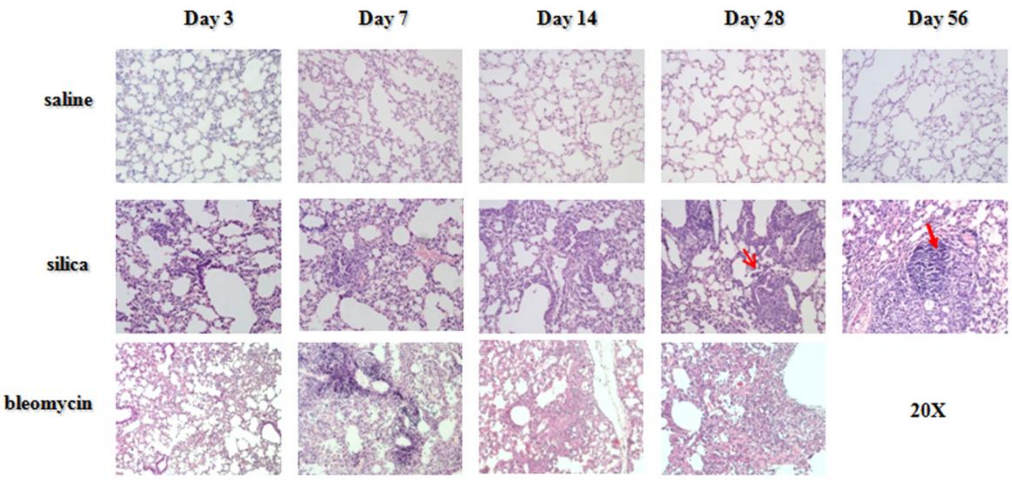

Figure S1. Photomicrographs showing HE staining (×200) of lung tissues after silica or bleomycin treatment. Mice were injected intratracheally with saline , silica (50 mg/kg) or bleomycin (1.5 U/kg) and sacrificed at days 0, 3, 7, 14, 28 or 56 after injection. There were no obvious abnormalities in the control group (day 0), the alveolar septum was destroyed from day 3 to day 14, the fibrotic cellular nodules were found at day 28 and fibrotic nodules were observed at day 28 and 56. n = 6 per group.

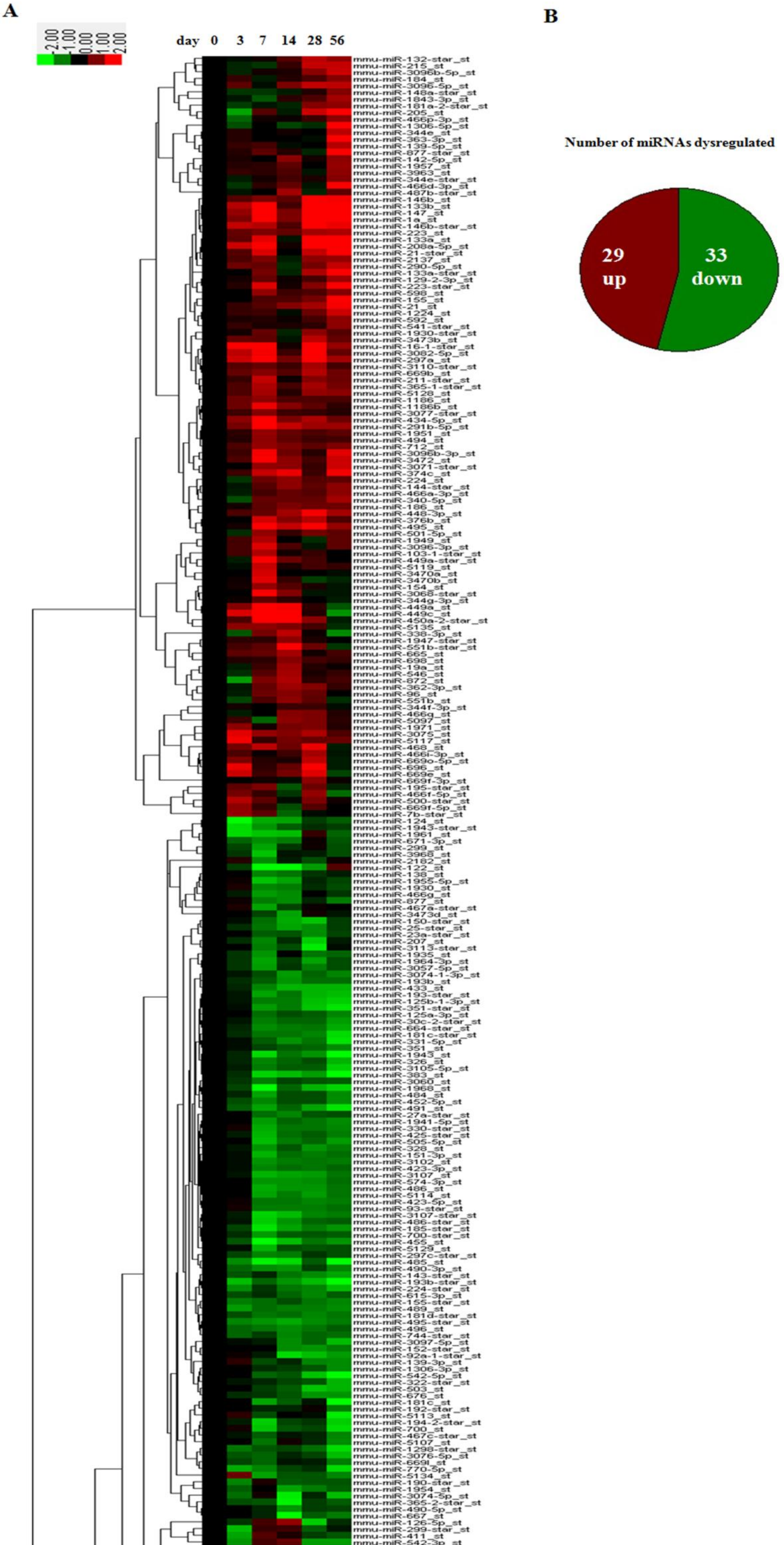
A
-2.00 -1.00 0.00 1.00 2.00
day 0 3 7 14 28 56
B
Number of miRNAs dysregulated
29 up
33 down

Figure S2. miRNA expression patterns in lung tissues of silica-induced pulmonary fibrosis model. **(A)** Hierarchical clustering algorithm of the GeneSpring software was used for clustering differentially expressed miRNAs according to their miRNA profiles. Two hundred and fifty-nine miRNAs altered at least twofold changes (in either direction) in at least one time point after treatment when compared to control (day 0). Up-regulated microRNAs are shown in progressively brighter shades of red, depending on the fold difference, and down-regulated microRNAs are shown in progressively brighter shades of green. **(B)** Venn diagram demonstrates 62 miRNAs altered at least 3 of the 5 comparisons, including 33 up-regulated (ratio ≥ 2) miRNAs and 29 down-regulated (ratio ≤ 0.5) miRNAs, compared to day 0.

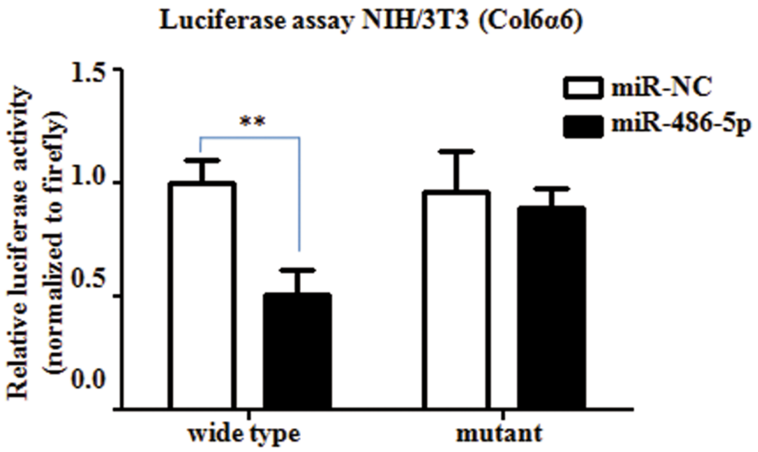

Figure S3. Col6a6 is a direct target of miR-486-5p. MiR-486-5p or miR-NC and human Col6a6 3’UTR-derived psiCHECK-2 construct (wild type or mutated in the

putative miR-486-5p seed region) were co-transfected in NIH/3T3 cells. Cells were harvested two days after transfection and luciferase activities were analyzed. All renilla luciferase activities were normalized with firefly luciferase activity. Values are presented as Means ± SD. Experiments were performed 3 times with similar results. ** $P < 0.01$.

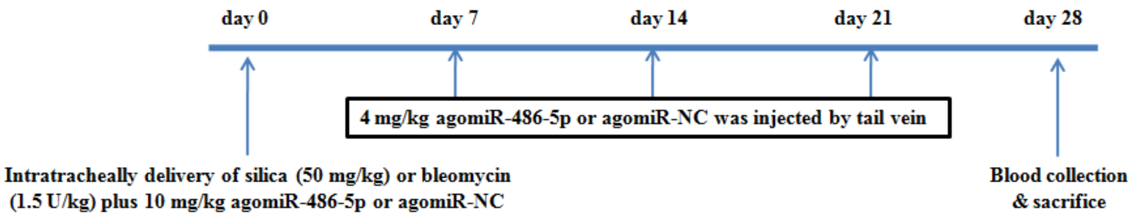

Figure S4. The paradigm of the experiment for miR-486-5p treatment. Control mimics plus silica/bleomycin (10mg/kg agomiR-NC plus 50 mg/kg silica or 1.5 U/kg bleomycin in 50 μl saline), miR-486-5p mimics plus silica/bleomycin (10mg/kg agomiR-486-5p plus 50 mg/kg silica or 1.5 U/kg bleomycin in 50 μl saline) were instilled intratracheally. Following this, 4 mg/kg agomiR-486-5p or agomiR-NC was injected by tail vein per week (n = 6 for each group). Mice were sacrificed 28 days after silica or bleomycin instillation.
